# ‘Walking epidural’: comparison of the analgesic efficacy of levobupivacaine 0.0625% + fentanyl 2mcg/mL versus ropivacaine 0.075% + fentanyl 2mcg/mL

**DOI:** 10.1186/s12871-023-02222-w

**Published:** 2023-08-01

**Authors:** Álvaro Mingote, Eloísa Zamora Moreno, Andrés García Díaz, Guillermo Chiara Graciani, Carlos Elbal Sánchez, Cristina Guadalix Sánchez, Diego Gutiérrez Martínez, Javier García - Fernández, Inocencia Fornet Ruiz

**Affiliations:** 1 Anesthesia, Critical Care Department and Pain Unit, Puerta de Hierro Universitary Hospital, Majadahonda. c/Manuel de Falla, 1, Madrid, 28222 Spain; 2 Faculty of Medicine, Autonomous University of Madrid, Madrid, Spain

**Keywords:** Walking epidural, Levobupivacaine, Ropivacaine, Fentanyl, Labor

## Abstract

**Introduction:**

Epidural infusion with low local anesthetic concentrations with opiates decrease the severity of the motor blockade associated. The present study aims to compare the analgesic efficacy and the motor blockade between two local anesthetic epidural infusions: levobupivacaine 0.0625% + fentanyl 2mcg/mL versus ropivacaine 0.075% + fentanyl 2mcg/mL.

**Materials and methods:**

In a single-blind prospective randomized study, 60 laboring parturient had continuous epidural analgesia as follows: 30 of them received levobupivacaine 0.0625% + fentanyl 2mcg/mL and 30 of them received ropivacaine 0.075% + fentanyl 2mcg/mL and rates of infusion were adjusted to the height. Analgesic, motor blockade and satisfaction records were collected as well as maternal and neonate adverse events.

**Results:**

After 2 h of the catheter placement, patients who received levobupivacaine showed a mean VAS of 3.2 [1.8–4.6] versus 1.8 [1.2–2.5] (*p = 0.05*) in patients who received ropivacaine. In addition, patients who received levobupivacaine showed a punctuation in Bromage scale of 0.0 [0.0–1.0] versus 0.0 [0.0–0.0] (*p = 0.04*) in patients who received ropivacaine. Finally, the parturient who received levobupivacaine scored a mean satisfaction index of 8.1 [7.3–8.9] versus 9.3 [8.7–9.8] (*p = 0.02*) in those who received ropivacaine. We did not register maternal nor neonate adverse events.

**Conclusion:**

Both infusions (levobupivacaine 0.0625% + fentanyl 2mcg/mL and ropivacaine 0.075% + fentanyl 2mcg/mL) are effective for labor analgesia. However, ropivacaine would present a better pharmacodynamic profile with less motor blockade and decreased need for analgesic rescue hence improving patient’s satisfaction.

## Introduction

Epidural technique was first described in 1921, when Spanish surgeon Fidel Pagés proposed the epidural space as the route for anesthesia (metameric anesthesia) for 43 patients [1]. Since then, central nervous system (CNS) blockades, especially epidural analgesia (EA), are considered the gold standard and the most employed technique for pain control during labor [2]. Currently, in addition to the reduction of pain, more women aim to feel their uterine contractions as well as to preserve the ability to walk and push during the expulsive process. Hence, currently the main objective of analgesic techniques for labor should emphasize on obtain analgesia as well as minimizing the motor blockade [3].

There are numerous studies comparing the analgesic efficacy of the different local anesthetics (LA) employed for epidural analgesia, as well as their dosage and their rate [4, 5]. It has been studied also the impact of their association to opiates because they would improve the quality of analgesia and reduce the dosage of LA during labor [6]. Hence, very low LA concentration associated to opiates would preserve the parturient ability to walk as the decrease in the dosage of LA diminishes motor blockade associated to EA. This EA technique has been commonly named as “*walking epidural”* [7].

The present study aims to compare the analgesic efficacy and the motor blockade between two local anesthetic epidural infusions: levobupivacaine 0.0625% + fentanyl 2mcg/mL versus ropivacaine 0.075% + fentanyl 2mcg/mL, as well as the maternal and neonate adverse events related to the technique.

## Materials and methods

### Description of the study and ethical approval

The present study is a prospective, single blind, observational comparative study. Ethical approval for this study was provided by the Ethical Committee for Medical Research of Puerta de Hierro Majadahonda Universitary Hospital (Madrid, Spain) (CEIm: 74/21) and meets the Declaration of Helsinki criteria. Written informed consent was obtained from all subjects.

### Patient selection

60 parturient who accomplished the inclusion criteria were included in the study (Table 1). The inclusion criteria were the following: age between 18 and 40 years, gestational age > 37 weeks, unique fetus, longitudinal situation and cephalic presentation, spontaneous labor with cervix dilation > 2 cm and < 5 cm. Exclusion criteria were the following: patient with pre-eclampsia or cardiac disease (NYHA III-IV), diabetes mellitus type I prior to the pregnancy, BMI > 40, multiple pregnancy, podalic presentation or induced labor.

### Epidural technique

The patient was positioned sitting on the bed to achieve an adequate exposition of the lumbar spine. Then, skin was cleaned with alcoholic clorhexidine and then a sterile field was prepared. L3/L4 space was identified by palpation through the Touffier’s line, and 4 mL of subcutaneous LA (lidocaine 2%) was administered in the puncture zone with a fine needle (23G). Tuohy’s needle (18G, 80 mm) was then introduced and fixed by supra and interspinous ligaments. The epidural space was then identified through the loss-of-resistance technique with a 10 mL low resistance syrinx filled with saline solution. An epidural catheter (closed tip with 3 lateral openings) was then introduced and a test dose consisting of 3 mL of 2% lidocaine + epinephrine 1: 200:000 was administered through the catheter to exclude intravascular or subarachnoid position of the catheter. The catheter was left with 3.5 cm of margin inside the epidural space.

Each patient received randomly an epidural infusion consisting of levobupivacaine 0.0625% + fentanyl 2mcg/mL or ropivacaine 0,075% + fentanyl 2mcg/mL. The initial volume of the bolus varied depending on the height of the patient: patients smaller than 160 cm received 8 mL, between 160 and 170 cm received 10mL and taller than 170 cm received 12 mL. Then, a continuous infusion was programed, and the rate was defined again by the same distribution of patient’s height: 10, 12 and 15 mL/h respectively. Finally, a patient-controlled rescue bolus of 10 mL of the same infusion was available every time, with a closing time of 20 min and a maximum of 3 boluses per hour.

### Data collection

Demographic data from the patients were collected at the beginning of the study: age, height, weight, medical and obstetrical history, gestational age. This data was analyzed, and no differences were found between groups (Table 1).

Since the beginning of the infusion (15 and 30 min later), and then every 2 h, the following data was recorded: visual analogic scale (VAS: effective analgesia was considered as declaring a mean VAS score less than 4 out of 10), Bromage scale, MRC (medical research council) scale, sensory level. After the completion of the labor process, we recorded total dosage of LA as well as the number of rescue bolus administered by the patient, the lateralization of the sensory level and VAS during expulsive phase. Eventually, we registered the characterization of the expulsive phase (instrumentalization, cesarean rate) as well as the maternal adverse events. We also collected Apgar scale at 1, 5 and 10 min from the birth of the neonate, as well as their blood pH. Finally, we registered parturient satisfaction (total satisfaction: 10 points), which ranges from 0 to 10 points depending on the level of satisfaction expressed.

### Statistical analysis

Sample size and power calculations were conducted before data collection. They were based on previous data from similar studies and determined using the Granmo 7.12 software program (Institut Municipal d’Investigació Mèdica, Spain), accepting an alpha risk of 0.05 and beta risk of 0.20 in a two-sided test, with an estimated common standard deviation of 1.3 points over the mean when it comes to VAS, priorizing to detect statistically significant difference greater than or equal to 1 units. Hence, 30 patients per group deemed adequate. This software is online and public. We describe those parameters that were not parametric as median [p25, p75] and those who were parametric as mean [IC_95%_]. Qualitative data are shown as percentage of the group (%).

For the analysis of quantitative variables, a normality test (Kolmogorov Smirnov test with Dallar – Willkinson – Llilliefor p value) was carried out. If the data distribution followed the normal distribution, a t test was performed to check for differences between groups. However, if they did not follow a normal distribution, a Mann Whitney test was performed to check for differences between groups. Statistical analysis was carried out using Prism Graphpad version 8.0 software. Statistical significance was assumed for two-tailed p < 0.05. To check for differences between qualitative variables, a chi-square test with Fisher’s correction was performed.

## Results

Main characteristics of the patients included in the study are shown in the Table 1: we did not find differences between groups in term of age, tall, weight, cervix dilation at epidural administration, sensory level at 30 min after epidural administration and deambulation time after epidural administration (Table 1).

**Table 1:** Basal characteristics of the patient included in the present study

	Ropivacaine group	Levobupivacaine group	P-value
**Patients (n)**	30	30	-
**Age (years)**	30 [32–37]	30 [31–38]	*P* = 0.48
**Tall (cm)**	166.3	164.3	*P* = 0.38
**Weight (kg)**	69.1	68.8	*P* = 0.83
**Cervix dilation at epidural administration (cm)**	3 [2, 3]	3 [3]	*P* = 0.23
**Primiparous (%)**	45	58	*P* = 0.37
**Sensory level 30 min after epidural administration**	T10 [T9 – T10]	T10 [T9-T10]	*P* = 0.71
**Deambulation time (mins)**	63 [34–92]	66 [26–102]	*P* = 0.88

### Analgesia

**Fig. 1:**
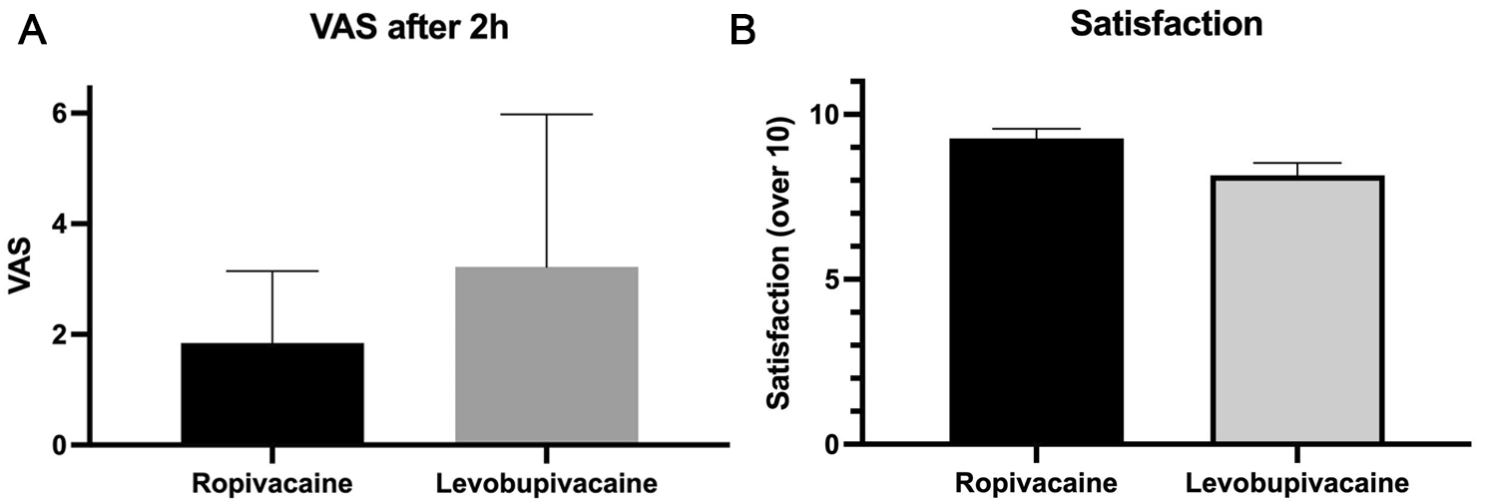
**A.** Mean VAS after two h of epidural administration (p = 0.05).) **B.** Mean satisfaction of the patients after the labor (p = 0.02)

The parturient that received the levobupivacaine infusion showed a mean VAS after 30 min of 1.1 [0.4–1.9] versus 0.9 [0.4–1.5] (*p = 0.7*) from those who received ropivacaine. After 2 h, patients who received levobupivacaine showed a mean VAS of 3.2 [1.8–4.6] versus 1.8 [1.2–2.5] (*p = 0.05*) in patients who received ropivacaine (Fig. 1-A). After 4 h, patients who received levobupivacaine showed a mean VAS 1.1 [0.3–1.9] versus 0.9 [0.4–1.5] (*p = 0.18)* in patients who received ropivacaine. Finally, during the expulsive phase, the levobupivacaine group showed a mean VAS of 2.9 [1.8–4.0] versus 2.2 [1.0–3.4] (*p = 0.21)* in the ropivacaine group.

### Motor blockade

After 30 min from the beginning of the epidural infusion, patients who received levobupivacaine showed median punctuation in Bromage scale of 0.0 [0.0–0.5] versus 0.0 [0.0–0.0] (*p = 0.97*) in the ropivacaine group. After 2 h, the levobupivacaine group showed a median Bromage of 0.0 [0.0–1.0] versus 0.0 [0.0–0.0] (*p = 0.04*) in the ropivacaine group. These results were the same after 4 h.

### Satisfaction and LA consumption

Levobupivacaine group required a mean ratio of rescue bolus of 0.6 bolus per hour [0.3–1] versus 0.3 bolus per hour [0.2–0.4] (*p = 0.05*) in the ropivacaine group. Total consumption of LA was 119.8 mg [81–156 mg] of levobupivacaine vs. 90.6 mg [67–113 mg] of ropivacaine. The levobupivacaine group showed a mean satisfaction index of 8.1 [7.3–8.9] versus 9.3 [8.7–9.8] (*p = 0.02*) in the ropivacaine group (Fig. 1-B).

### Maternal complications

During labor, 47% of the patients from the levobupivacaine group required the administration of a rescue bolus with a higher concentration of LA, versus 17% (*p = 0.05*) in the ropivacaine group. Mean duration of labor was 7.1 h [5.4–8.7] in the levobupivacaine group versus 5.7 h [4.2–7.2] (*p = 0.26*) in the ropivacaine group. We did not register any complications due to the epidural technique and cesarean rate in the levobupivacaine group was 7.2% versus 6.5% in the ropivacaine group (*p > 0.6*). We did not register nausea, vomiting or pruritus episodes due to the epidural infusion (in fact, lower LA concentrations would decrease maternal adverse events).

### Neonate complications

Mean pH from neonates whose mother received levobupivacaine was 7.27 [7.25–7.28] versus 7.25 [7.21–7.29] in those whose mother received ropivacaine (*p = 0.25*). Median Apgar of the levobupivacaine group after 5 min from birth was 10 [10] versus 9.5 [9, 10] (*p > 0.6)* in the ropivacaine group. No neonatal complications (bradycardia or low Apgar scores) were registered.

## Discussion

In our study we have observed that levobupivacaine and ropivacaine in infusions at lower concentrations than traditionally considered [8, 9] are effective and safe for labor analgesia. Both LA infusions provide effective analgesia (VAS < 4) and in both groups the patients showed a minimum or even no grade of motor blockade.

However, we have also observed that these effects are not equivalent, but they differ in terms of intensity of motor blockade and analgesic efficacy. It seems that ropivacaine produced an increased dissociative analgesia (increased analgesia and lower motor blockade) than levobupivacaine. This fact would result from the lower liposolubility of the ropivacaine, which would have a lower penetration on richly myelinated motoneuron fibers, otherwise known as type A fibers [10, 11]. This matter was indeed studied more than 20 years ago [8], although we did not find other studies that employed such lower concentrations of LA that we used. In addition, other studies that compare epidural infusion of LA do not include opiates on the infusion due to a possible overlapping in terms of analgesic efficacy attributed to LA [12].

Traditionally, ropivacaine has always been considered as less potent than levobupivacaine (at least in analgesic terms), which has led to studies that claim that with ropivacaine infusions, rescue boluses are more requested [8]. In contrast, we have observed that equating both concentrations to analgesic potency of each LA (based on the pharmacokinetic profile [13]), results in patients requiring half of rescue boluses with ropivacaine than with levobupivacaine, suggesting that ropivacaine, at equipotent concentrations would produce more analgesic quality than levobupivacaine in epidural infusions.

Furthermore, safety of both LA during labor have been studied and it have been observed that ropivacaine would present a quicker onset and lower hemodynamic adverse events, lowering cesareans rate [14]. However, other, and older studies suggest that ropivacaine would produce more adverse events [8]. In this sense we have not observed significant differences when it comes to clinical hemodynamic complications such as maternal clinical hypotension between both groups (perhaps due to the use of lower doses of LA) and although we have seen a lower cesarean rate in the ropivacaine group, this gap was not statistically significant, and we doubt of its clinical relevancy.

In addition, we did not register nausea, vomiting or pruritus episodes due to the epidural infusion (in fact, lower LA concentrations would decrease maternal adverse events). No neonatal complications (bradycardia or low Apgar scores) were registered.

However, the present study has some limitations. First, our patients were from the same hospital and perhaps our results might not be extensible to the general population due to socio-cultural influences. Finally, we did not manage to obtain statistical significancy when we evaluated variables corresponding to a labor duration superior to 4 h, perhaps because a considerable proportion of each group terminated their labor before 4 h, hence reducing our statistical potency.

## Conclusion

In the present study we observed that, although both LA infusions (levobupivacaine 0.0625% + fentanyl 2mcg/mL versus ropivacaine 0.075% + fentanyl 2mcg/mL) are effective for epidural analgesia during labor, ropivacaine would present a better pharmacodynamic profile as it produces a better analgesia, lower motor blockade and a better satisfaction of the patient, which would be probably in relation to its pharmacokinetic profile. In addition, we did not registered any adverse event associated to any of the LA infusions.

## Data Availability

The datasets used and/or analysed during the current study are available from the corresponding author on reasonable request.
